# Refractory Celiac Disease: What the Gastroenterologist Should Know

**DOI:** 10.3390/ijms251910383

**Published:** 2024-09-26

**Authors:** Mariana Verdelho Machado

**Affiliations:** 1 Gastroenterology Department, Hospital de Vila Franca de Xira, 2600-009 Lisbon, Portugal; mverdelhomachado@gmail.com; Tel.: +351-912620306; 2 Gastroenterology Department, Faculdade de Medicina, Lisbon University, 1649-028 Lisboa, Portugal

**Keywords:** non-responsive celiac disease, refractory celiac disease, aberrant intraepithelial lymphocytes

## Abstract

Fewer than 1% of patients with celiac disease (CD) will develop refractory CD (RCD). As such, most gastroenterologists might never need to manage patients with RCD. However, all gastroenterologists must be familiarized with the basic concepts of RCD and non-responsive CD (NRCD), since it can present as a severe disease with high mortality, not only due to intestinal failure, but also due to progression to enteropathy-associated T cell lymphoma (EATL) and a higher susceptibility to life-threatening infections. The diagnostic workup and differential diagnosis with other causes of gastrointestinal symptoms and villous atrophy, as well as the differentiation between type I and II RCD, are complex, and may require specialized laboratories and reference hospitals. Immunosuppression is efficient in the milder RCDI; however, the treatment of RCDII falls short, with current options probably only providing transient clinical improvement and delaying EATL development. This review summarizes the current diagnostic and therapeutic approach for patients with RCD that all doctors that manage patients with CD should know.

## 1. Introduction

Celiac disease (CD) is a systemic disease triggered by an immune response to ingested gluten in susceptible patients. It roughly affects 1% of the population [1], and presents with an enteropathy characterized by villous atrophy and intraepithelial lymphocytosis, as well as the presence of specific serum autoantibodies such as IgA anti-transglutaminase-2 (TTG) and anti-endomysium (EMA) [2].

The only approved treatment for CD is a strict gluten-free diet (GFD) [3]. Adherence to a GFD results in an improvement in symptoms after 1 month and complete resolution in 6 months [4,5]. However, in up to 50% of CD patients on a GFD, symptoms persist after 6 to 12 months, which is designated as non-responsive CD (NRCD) [6]. A GFD also results in seroconversion to negative TTG in more than 80% of the patients in 6 months. Nevertheless, in adults, mucosal histologic recovery is delayed and not universal [7], with only one third of the patients presenting normal villous architecture in 2 years, two thirds in 5 years and 90% at 9 years [8,9,10]. Indeed, 30–40% of the patients are slow responders, taking more than 5 years to achieve histologic complete remission [11,12].

Refractory CD (RCD) is defined as the persistence of symptoms of malabsorption (diarrhea, weight loss, anemia or nutritional deficiencies), associated with villous atrophy, after 1 year on a GFD [13], in the absence of other disorders including overt lymphoma [14]. For an RCD diagnosis, it is advisable to demonstrate the presence of the HLA-DQ2/8 haplotypes and of positive serology at CD presentation [13]. It is a rare complication of CD, virtually only occurring in adults [15]. Older studies presented higher prevalences, of up to 10%, of RCD among patients with CD, while more recent studies showed prevalence lower than 1% [15,16,17,18,19,20,21,22,23], suggesting a decrease in the prevalence of RCD complicating CD [24]. Most CD patients with persistent symptoms and/or villous atrophy on a GFD, present a different causal diagnosis, the most frequent one, in up to 50% of the cases, being gluten contamination of the diet [18,25]. Indeed, a diagnosis of RCD can only be made in 10–20% of patients with NRCD [18,26].

RCD tends to occur early, but can occur at any time after the diagnosis of CD [27].

RCD can be subdivided in two very different entities, type I RCD (RCDI) and type II RCD (RCDII) (Table 1).

RCDI seems more prevalent than RCDII, accounting for 60–90% of RCD cases in most series [20,21,23,28,29], even though two European studies described a predominance of RCDII [30,31]. RCDI is usually a mild condition, clinically and histologically similar to active uncomplicated CD [28,31], with a 5-year survival of 80–90% [28,30,31,32]. On the contrary, RCDII is usually a severe condition, with extensive intestinal mucosal damage and the presence of phenotypically aberrant intraepithelial lymphocytes (IEL), with a 5-year survival less than 50% [28,30,31,33], mainly due to a high risk of progression to enteropathy-associated T-cell lymphoma (EATL), intestinal failure and infections [29]. RCDII is considered a low-grade intraepithelial lymphoma or in situ lymphoma [6,34].

This review will summarize the clinical approach for the diagnosis and treatment of RCD.

## 2. Pathogenesis

### 2.1. Risk Factors for RCD

RCD is a rare condition, and it is believed to be associated with strong anti-gluten responses and long-term exposure to gluten [30]. In accordance, older age at CD diagnosis is consistently a risk factor for RCD, increasing two-fold after 40 years and 18-fold after 60 years of age [15]. Classic presentation [15] and worse symptoms including weight loss and diarrhea also associate with an increased risk for RCD [23], and more than half the patients present primary unresponsiveness to a GFD [35]. Furthermore, compared to uncomplicated CD, patients with RCD tend to present a longer delay in CD diagnosis before the onset of symptoms [24]. This suggests a long-term exposure and a strong immune reaction to gluten [36] previous to the first manifestation of CD, that already presents with GFD refractoriness. Furthermore, HLA-DQ2 homozygosity, indicating poor prognosis, albeit being similarly frequent in uncomplicated CD and RCDI, increases three-fold the risk for RCDII and four-fold for EATL [37].

Infections, particularly viral infections, have been hypothesized as an environmental factor that could predispose to RCD, promoting gut inflammation and injury as well as CD8+T-cells survival and proliferation mediated by type I interferon-induced or toll-like receptor (TLR)-3-induced interleukin (IL)-15 overexpression [38,39].

### 2.2. Pathological Processes in RCDI

The pathogenesis of RCDI is still to unravel, and, probably, RCDI encompasses different mechanisms of GFD refractoriness [11]. One such mechanism could be the development of a collagenous sprue leading to persistent symptoms despite a GFD, which has been described in RCDI patients [40]. More recently, an association between collagenous sprue and RCDI has been questioned [41]. The most appealing hypothesis is that patients with RCDI shifted their intestine immune reaction from gluten-induced into a gluten-independent autoimmune reaction [6,11]. Supporting this hypothesis is the fact that patients with RCDI, as compared to patients with uncomplicated CD, present a tendency to a 50% higher prevalence of other associated autoimmune disorders [20], as well as the good response to immunosuppressive treatment [13]. IL-15, overexpressed in some RCDI patients, could play a role, by promoting survival of autoreactive T cells [42].

### 2.3. Pathological Processes in RCDII

RCDII is a completely different entity, being currently understood as an intraepithelial low-grade lymphoma [43]. It is characterized by the presence of aberrant IELs that can be viewed as neoplastic cells, even though being cytologically normal and with a low proliferative index [6]. Aberrant IELs are a distinctive set of cells that share features of T lymphocytes and of natural killer (NK) cells. Indeed, aberrant IELs do not express surface T-cell, B-cell nor NK-cell lineage markers, respectively, CD3 or T-cell receptor (TCR), CD19 and CD56. However, they do express cytoplasmic T-cell lineage CD3 and did undergo clonal rearrangement in the TCR gene (TR), suggesting early T-cell commitment that was interrupted during cell differentiation (hence failing to achieve full T-cell differentiation) and shifted to a NK commitment, expressing NK receptors such as NKp46 [44,45]. This shift in lineage differentiation programming seems to be the consequence of sequential NOTCH and IL-15 signaling. NOTCH signals in the intestinal mucosa first direct IELs towards T-cell differentiation, with a change in reprogramming towards a NK-like phenotype (from adaptive to innate-like cells) through IL-15 stimulation. These cells might have a protective role in the intestinal mucosa, producing interferon-γ and exhibiting NK-like cytotoxic activity.

In the normal intestinal mucosa, a set of IELs (on average, 8%, but up to 20%) express cytoplasmic but not surface CD3, seem to derive from hematopoietic precursors that undergo extrathymic differentiation into either T or NK cells [46] and are thought to be the precursors of aberrant IELs [47]. In RCDI, those intermediate cells correspond to less of 5% of IELs, even less than in the normal mucosa, probably due to the expansion of T CD8+ IELs [31]. In RCDII patients, a massive overexpression of IL-15 [48,49] promotes the expansion of aberrant IELs, through decreased apoptosis/increased survival and not through increased proliferation [44]. IL-15 impairs aberrant IELs apoptosis through janus kinase 3 (JAK-3), transcription factor STAT-5 and the anti-apoptotic Bcl-xL pathway [50]. Expanded aberrant IEL can mediate an intense cytolytic attack of the gut epithelium, explaining the severe mucosal injury with the development of ulcerative jejunoileitis and the extreme malabsorption phenotype [51].

Aberrant IELs accumulate chromosomal abnormalities and several somatic oncogenic mutations. Indeed, 80% of RCDII patients present IELs somatic gain-of-function mutations in the kinase JAK-1 or its downstream effector STAT-3, which can provide a selective advantage for aberrant IELs expansion [52]. This pathway has a major role in promoting lymphocyte proliferation, survival and activation, being triggered, among others, by interferon-γ and IL-15 [6]. One particularly frequent mutation, present in 50% of RCDII patients and 68% of EATL cases complicating RCDII, is the JAK1 p.G1097 [53], a hotspot mutation localized in a highly conserved position at the site of interaction of the JAK-1 negative regulator, a suppressor of cytokine signaling (SOCS)-1 [54].

**Figure 1:**
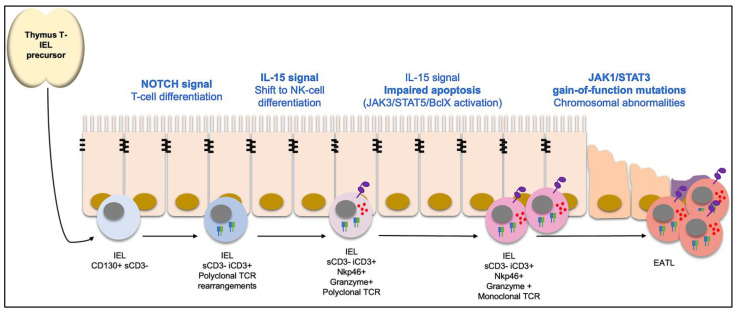
Current hypothesis for RCDII pathogenesis. Thymus-originating intraepithelial lymphocytes (IEL) precursors commit to T-cell differentiation (expressing cytoplasmic CD3 and undergoing T cell receptor (TCR) rearrangements) in response to NOTCH signals. This differentiation is interrupted and shifted towards a NK-cell differentiation (expressing granzyme and NK receptors such as NKp46) in response to Interleukin-15 (IL15). IL15 promotes the accumulation of those IELs through JAK3/STAT5/BclX inhibition of apoptosis. Aberrant IELs are highly cytotoxic, promoting extensive mucosal damage. Also, those cells acquire mutations, for example, in the JAK1/STAT3 pathway, that further increase their survival advantage. Further on, additional somatic mutations drive the development of enteropathy-associated T-cell lymphoma (EATL).

Up to 50% of RCDII patients develop EATL, usually in the first 18 months [30]. The aberrant IELs seem to be the precursor cells in EATL, sharing the same phenotype, TR monoclonal rearrangement [55,56,57] and chromosomal abnormalities/oncogenic somatic mutations [53]. Indeed, 90% of EATL patients in the context of RCDII present the same JAK1/STAT3 mutations described in RCDII [53]. Longitudinal studies showed that RCDII patients that do not progress to EATL tend to maintain stable mutation profiles, whereas the ones who progressed to EATL acquired additional mutations [58] (Figure 1).

Interestingly, cells phenotypically similar to aberrant IELs can also be found in the lamina propria, other gastrointestinal epithelium tissues, blood and other organs such as the skin and lungs [59,60,61], explaining why EATL does not necessarily develop in the intestine, and can arise in different organs such as the skin [11].

## 3. Diagnosis

### 3.1. Is It RCD?

When faced with a patient with NRCD, with persistent or recurrent symptoms despite a GFD for 6–12 months, the first attitude should be to review the previous diagnosis of CD, reviewing the serology at diagnosis, duodenal histology and presence of HLA-DQ2/8 [13].

Afterwards, contamination of the diet with gluten should be excluded [14], as it is responsible for persistent symptoms and mucosal damage in 40–50% of NRCD patients [18,25,26]. It is difficult to adhere to a strict GFD, since it is highly widespread, with a typical western diet providing 15–20 g of gluten per day [62], whereas 50 mg of gluten ingestion (which corresponds to only 1/100 of a slice of bread) is the demonstrated threshold to induce mucosal damage [63]. Gluten is exceedingly used in the food industry, since its elastic properties provide an appealing texture to food, being present in implausible products such as yogurt, frozen fish and ice-creams [64,65], as well as non-dietary sources such as in the excipient of medications [66], toothpaste and lipstick [67]. A GFD imposes challenges in the daily life of patients, for example, when dining out, travelling, and socializing with peers [68], so much so that the perception of CD patients on the negative burden of treatment/GFD is similar to the one expressed by patients with chronic kidney disease on hemodialysis [69]. The aggregate data explain why 40–90% of CD patients do not adhere to a strict GFD [70]. Noteworthy, even in patients proclaiming to adhere to a strict GFD, it is possible to demonstrate inadvertent or intentional gluten consumption in 70–80%, when evaluated by food questionnaires [71] or by the presence of immunogenic gluten peptides in the urine [72,73,74], with an average consumption of 150 mg [75], that is, three times the threshold for triggering mucosal damage [63]. Serology (mainly TTG) can help, since when positive has a high specificity to gluten intake [76], with TTG titers correlating with the severity of the transgression [77]. However, a negative title does not exclude gluten contamination [5], since its sensitivity is only 50% [27]. Furthermore, a caveat is that in RCD, with persistent mucosal inflammation, serology can remain positive despite a GFD [13].

**Figure 2:**
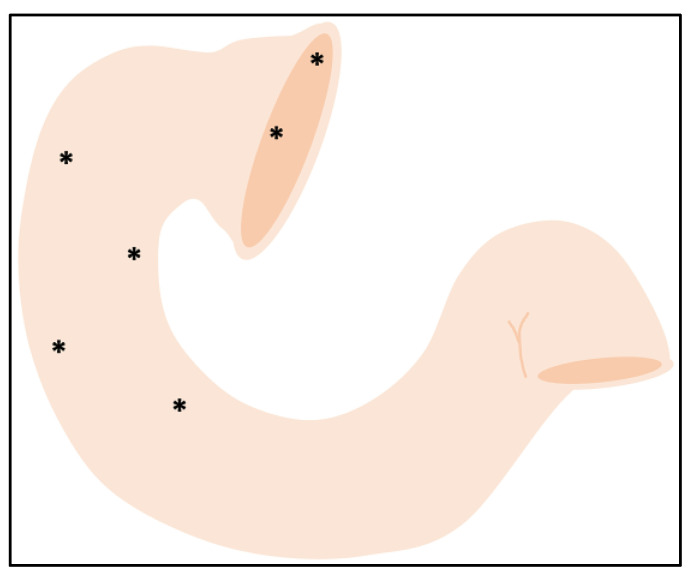
Duodenal biopsies protocol for RCD. * represent the recommended biopsy sites.

The next step should be to perform an upper endoscopy with duodenal biopsies to assess mucosal damage, that is, if villous atrophy persists (pVA). The protocol for collecting duodenal biopsies should be the same proposed for CD diagnosis, that is, at least four biopsies from the second part of the duodenum and one to two biopsies from the duodenal bulb at the 9 and 12 o’clock positions [13,78,79,80], since this increases, up to seven times, the accuracy for pVA [81], due to the uneven distribution of mucosal damage [82] (Figure 2).

Bulb and duodenal biopsies should be sent in separate containers so villous atrophy is not overrated, since bulb villi are shorter, and the evaluation of villous architecture in the bulb may be hampered by the presence of Brunner glands, lymphoid tissue, peptic duodenitis or gastric metaplasia [83,84]. Many gastroenterologists recommend obtaining a single biopsy specimen with each pass of the forceps, in order to improve the specimen orientation [85] and avoid sample lost [86], even though a recent study was not able to demonstrate that single biopsies would increase the accuracy for detecting villous atrophy [87]. If possible, 2–3 extra biopsies should be collected and sent in saline or RPMI to flow cytometry studies, alongside two more for T-cell receptor (TCR) gene rearrangement studies [13].

If duodenal villous architecture is normal, other conditions should be ruled out, such as irritable bowel syndrome, that can coexist with CD, explaining more than one fifth of the cases [88], lactose intolerance [89], small intestinal bacterial overgrowth [90], pancreatic exocrine insufficiency [91,92,93], and microscopic colitis (CD patients have a 10-fold increased risk of microscopic colitis, and up to a 35-fold increase in the first year after CD diagnosis [94]). 

In the case of pVA, even more so when serology is negative, other causes of villous atrophy should be excluded [13] such as drug-induced enteropathy, infectious enteropathy, autoimmune enteropathy, common variable immunodeficiency (CVID), tropical sprue, Crohn’s disease and EATL [36].

Drug-induced enteropathy has been described for angiotensin receptor antagonists (ARA), particularly olmesartan [95], immunomodulators such as mofetil mycophenolate, methotrexate and azathioprine [96] and checkpoint inhibitors [97]. Olmesartan-associated enteropathy was first described in 22 patients from the Mayo Clinic, with negative TTG serology, who did not respond to a GFD, but did respond clinically and histologically to the suspension of olmesartan, after an average of 8 months [98]. Afterwards, other patient cohorts were described in France [99,100], though Korean large-scale epidemiologic studies did not confirm an association between olmesartan and an increased risk for enteropathy [101], suggesting that genetic susceptibility might predispose to olmesartan-induced mucosal damage. The long delay between the onset of olmesartan prescription and the development of enteropathy (which can take months to years) suggests a cell-immunity mediated injury [100]. The risk for enteropathy increases with a longer time of exposure [102], increasing more than 10 times after 10 years [103]. The enteropathy risk is not an ARA class effect, with olmesartan presenting a particularly high risk, and only a few cases described for irbesartan [99]. Histology can give us some cues to suspect this diagnosis, with relevant granulocytic infiltration (neutrophils and eosinophils) and increased crypt apoptosis [102]. Checkpoint inhibitors can also induce an immune-mediated enteropathy with villous atrophy [97], being described even in cases of CD diagnosed after starting therapy [104,105]. A histologic cue to differentiate from CD is the presence of neutrophilic infiltration and/or erosions. Enteropathy can occur any time during therapy and even months or years after termination [97].

Infectious enteropathy should be excluded, such as *Giardia lamblia* [106], tuberculosis and other Mycobacteria [107], Whipple’s disease and human immunodeficiency virus-associated enteropathy [108].

Autoimmune enteropathy typically affects children, commonly infants in the first 6 months of life, but it can also occur in adults [109]. It manifests with severe diarrhea and malabsorption, frequently in association with other extraintestinal autoimmune conditions. The presence of anti-enterocyte antibody targeting the brush border protein harmonin is highly suggestive of autoimmune enteropathy [110], though inconstant, and up to 30% of the patients may present IgA TTG, even though with negative EMA [111]. Histologic differences from RCD are the lower extent of intraepithelial lymphocytosis presenting both CD8+ and CD4+ cells, in contrast with a prominent lamina propria T-cell infiltration and villitis (that is, intraepithelial neutrophils), the absence of Paneth or goblet cells, the presence of crypt abscesses and glandular apoptosis [109,112]. Treatment may be challenging, with a low response to steroids, even though budesonide may be effective [109].

One third of CVID with gastrointestinal symptoms or anemia presents celiac sprue-like enteropathy, even though other causes of diarrhea such as chronic infection with *Giardia lamblia*, *Campylobacter jejuni* and *Salmonella* must be ruled out [113]. Distinctive duodenal histology features are the profound depletion of plasma cells and follicular lymphoid hyperplasia. It does not respond to intravenous immunoglobulin, nor to a GFD, but steroids are highly effective [113].

Lastly, tropical sprue should be suspected in residents or travelers to India, Southeast Asia and the Caribbean islands. In contrast with CD, Marsh changes are milder and there is a decrescendo type of intraepithelial lymphocytosis [114]. Treatment requires a combination of folic acid and tetracycline antibiotics [114,115].

### 3.2. Is It RCDI or RCDII?

After exclusion of other causes for villous atrophy, the diagnosis of RCD can be made, and the workup should proceed to differentiate RCDI from RCDII [13].

RCD tends to occur in older patients, typically older than 50 years old [6,28]. RCDI presents a female predominance (three quarters of patients), unlike RCDII (only one third to one fourth of patients are female) [28]. RCD can present as primary GFD non-response or after an initial response in half of RCDI patients and only one third of RCDII patients [29]. At diagnosis, RCDII patients show a worse clinical presentation, with abdominal pain being more frequent, alongside worse malnutrition and hypoalbuminemia [28,29,31]. RCDII patients also have a high risk of severe infections, even disseminated fungal infections [29]. Endoscopically, the extent of intestinal mucosal damage is wider, with distal small bowel involvement in more than half of RCDII patients, and less than 20% of RCDI patients [29,116]. Mucosal damage is worse, with ulcerative jejunitis (defined with the presence of ulcers of at least 1 cm diameter) in 67% of RCDII and only 10–29% of RCDI patients [28,31,116]. Indeed, in RCDI, small bowel erosions can occur, but large ulcers virtually indicate RCDII [116].

Histologically, RCDI is indistinguishable from active CD, even though it may display a collagenous sprue with a thickened subepithelial collagen band [40]. RCDII usually presents with severe mucosal damage, with subtotal or total villous atrophy and extensive intraepithelial lymphocytosis. Importantly, these lymphocytes, even though cytologically normal and with a low proliferative index, are phenotypically abnormal [6]. Aberrant IELs express markers of IEL such as CD103 (formerly called HML-1, a member of the integrin family αEβ7, receptor for epithelial E-cadherin, an adhesion molecule that allows interaction with enterocytes [117,118]), the panleukocyte CD45 and CD7 (a marker of thymocytes and mature T cells) [52]. However, aberrant IELs are lineage negative, that is, they do not express cell surface markers CD3, CD14, CD19, or CD56, nor T-cell receptor (TCR), but do express cytoplasmic CD3, which differentiates them from T cells, B cells, NK cells and lymphoid tissue inducer cells [11,119]. Aberrant IEL cells also lack CD8, CD4 or CD5 markers, but express NK receptors such as NKp46 [120]. Furthermore, aberrant IELs have undergone clonal rearrangement on the TCR gene, TR. It is the demonstration of aberrant IELs that allows the distinction between RCDII and RCDI. Those cells can be detected by immunohistochemistry or multicolor flow cytometry. Clonal TR rearrangement can also be evaluated by multiplex polymerase chain reaction (PCR). 

Regarding immunohistochemistry, antibodies against CD103, αβ or γδ TCR only work on frozen tissue sections. Furthermore, cytoplasmic CD3 cannot be differentiated from membrane CD3 by immunohistochemistry. As such, paraffin sections will show a high number of cells that express CD3, but do not express CD4 or CD8. However, in untreated or GFD-treated CD patients, up to 50% of IELs (on average 20%) are γδ TCR cells, the majority of which are CD8 negative, and do not constitute aberrant IELs [121]. Hence, the cutoff to diagnose RCD by immunohistochemistry is at least 50% of IELs being aberrant [76]. Although this technique is widely available, its performance in the diagnosis of RCDII is variable among centers and with suboptimal concordance [122], with a described sensitivity ranging from 62 to 100% and specificity from 70 to 100% [123]. A recent advance in immunohistochemistry is staining for NKp46, which, when present in more than 25 IELs per 100 enterocytes, seems to discriminate between RCDII and RCDI with a 95% positive and 87% negative predictive value [120].

The best technique to detect and quantify aberrant IELs is multicolor flow cytometry, with a 20% cutoff of aberrant IELs being proposed to differentiate RCDII from RCDI [57]. It is the reference technique recommended by current guidelines [13,76]. Flow cytometry not only can distinguish sCD3 (surface CD3) from iCD3 (intracytoplasmic CD3), by staining after cell permeabilization [57], but it can better characterize and quantify the different populations of lymphoid cells through the simultaneous evaluation of multiple antigens [6]. This technique first discriminates the intraepithelial localization of lymphocytes, attesting that they are indeed IELs, by the expression of CD103, and, afterwards, aberrant IELs are distinguished by presenting sCD3−, iCD3+, CD8− and αβTCR− [57]. The absence of CD30+ Ki67+ lymphocytes also helps rule out EATL [6]. However, flow cytometry is not widely available, and should only be performed in experienced laboratories that can evaluate small samples and interpret the results according to the state of the art [124].

Importantly, cells with the same phenotype as aberrant IELs can also be detected in subepithelial layers of the small intestine such as the lamina propria [60], in other gastrointestinal epithelium tissues (in lymphocytic gastritis and colitis) [59] and even in extra-gut localizations such as the blood, skin and lungs [59,60,61]. This does not indicate the presence of EATL [124].

The assessment of clonal rearrangements in the TR gene can also support the RCDII diagnosis [13,76]. It is performed by multiplex PCR in DNA extracted from paraffin-embedded samples or fresh biopsies. However, this technique is prone to false positives [125]. Indeed, clonal or oligoclonal TR rearrangements can be observed in up to 15% of RCDI and even in a small proportion of uncomplicated CD [125]. The determination of clonal TCR rearrangements has shown variable sensitivity (53–100%) and specificity (0–100%) [123]. The clonality analysis should be preferably performed for TRG (γ TCR chain), due to the later occurrence and lower frequency in TRB (β TCR chain) [126,127]. Clonal TRD rearrangements (δ TCR chain) have been described in cases lacking TRG rearrangements [45].

Lastly, even though not implemented in clinical practice, mutational analysis for the detection of JAK1 (for example, the mutation p.G1097, present in up to 50% of RCDII patients) and STAT-3 mutations [53,58] may be a supportive criterion for the diagnosis of RCDII [6].

### 3.3. Exclusion of Complications

Nutritional deficiencies should be evaluated in all patients, such as liposoluble vitamins (A, D, E and K), iron, folates, B12 vitamin, copper, zinc, thiamin, B6 vitamin, thiamine and selenium [13].

In patients with RCD, particularly RCDII, the extent of the small intestine should be evaluated, in order to exclude complications such as jejunoileitis and EATL [13]. This can be done by a complementary study with videocapsule endoscopy (VCE) and magnetic resonance enterography (MRE) [128]. VCE not only showed a high sensitivity (89%) and specificity (95%) to estimate the extension of villous atrophy [129], but, more importantly, it may detect ulcerative jejunoileitis and small bowel neoplasms [116,130,131], with a diagnostic yield of almost 50% in patients with complicated CD [132]. MRE can give us additional information such as thickening or mass-forming lesions from the small intestine wall, increased enhancement, bowel dilatation, intussusception and enlarged mesenteric lymph nodes [133]. Spleen atrophy is also more frequent in RCDII and EATL [14]. Double-balloon enteroscopy should be performed for histologic evaluation of stenosis or masses, inflammation, erosions or ulcers, since up to 40% of lesions are unreachable by upper endoscopy [13,132].

**Figure 3:**
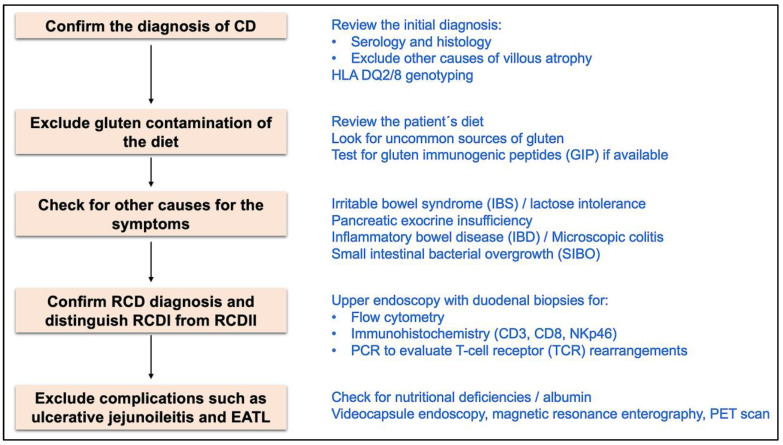
Diagnostic work-up for RCD.

In sick patients, a ^18^F-FDG PET may be useful to exclude EATL, with higher accuracy than computed tomography scan [134], since ^18^F-FDG does not appear to significantly accumulate in the gut of patients with CD, irrespective of disease activity [135]. EATL development occurs in up to 50% of RCDII patients (usually in the first 18 months) and in up to 14% of RCDI patients in the most pessimistic cohorts (which may be overestimated by misdiagnosis of RCD) [28,30,31] (Figure 3).

## 4. Treatment

Currently, we still lack a definitive treatment that is effective in eliminating aberrant IELs and able to prevent progression to EATL. Hence, available treatments are efficient for RCDI patients but fall short for RCDII patients.

### 4.1. Management of Complications

Patients with RCD should be submitted to a detailed nutritional assessment, including the determination of fat-soluble vitamins (A, D, E and prothrombin time as a surrogate for vitamin K deficiency), folate, iron, B12 vitamin, copper and zinc. Consider checking also for thiamine, magnesium, selenium and B6 vitamin determination. Albumin should be monitored as it is of prognostic value. Nutrition support and macro- and micronutrients deficiency should be supervised by a dietitian. Severe malnutrition and hypoalbuminemia should be addressed, as needed, with intravenous parenteral nutrition and albumin supplementation [13].

### 4.2. Drug Therapy

The level of evidence for the management of RCD is usually low, since it is a rare condition, resulting in only a few small randomized clinical trials evaluating it [136].

The first-line treatment for RCD is glucocorticoids [13]. The preferred one is open-capsule budesonide, which targets local inflammation, while avoiding systemic toxicity [6], due to the strong first-pass metabolism by cytochrome P450 in the liver [137]. The rationale for the open-capsule protocol is that enteric coated budesonide delivers the active drug to the distal small intestine and colon, while less than 30% is released in the proximal intestine [138], which is not concordant with the distribution of the mucosal damage in RCD [131]. The dose of budesonide should be 3 mg, three times a day. The morning capsule should be open and ground, the lunch dose should be open but not ground, and the evening capsule should be taken unopened [139], in order to allow delivery in the entire small bowel, including the duodenum and jejunum, but also the distal bowel [140]. The delivery to the entire bowel is important, since the distal component becomes increasingly affected in RCDI and even more in RCDII [131]. Patients should be advised to avoid foods such as grapefruit and drugs such as ketoconazole or oral contraceptives that impair cytochrome P450 function, and, hence, could abrogate the first-pass inactivation of budesonide, possibly allowing for systemic side effects [141]. Retrospective cohorts showed better responses with open-capsule- as compared to enteric-delivery budesonide [32,137,139,140,142]. In the largest study [140], on 43 RCDI and 13 RCDII patients, open-capsule induced a clinical response in 89% of patients (complete in two thirds) and a histological response in 89% (half to two thirds with complete response). Of interest, on follow up, in five of the 13 RCDII patients (38%), aberrant IELs were not detected by immunohistochemistry. However, the resolution of molecular and genetic abnormalities in RCD remains controversial and no long-term studies have demonstrated that open-capsule budesonide or steroids could eliminate aberrant IELs and prevent EATL [6,13,53]. Additionally, other cohorts even failed to demonstrate a histologic response in RCDII patients [139]. After clinical and histologic response, budesonide should be tapered by removing one pill every 3 months, the last dose being one pill every other day [140]. Importantly, after tapering, recurrence has been described in 25% to over 80% of cases [140,142].

Alternative glucocorticoids could be prednisolone and intravenous methylprednisolone in severe disease [13]. Patients that do not respond, present incomplete response or experience recurrence after tapering glucocorticoids are eligible for second-line therapies. The best second-line therapy is yet to be defined, and the level of evidence is low, relying on small retrospective cohorts. For patients with RCDI, second-line therapies should be immunomodulators, such as azathioprine, mercaptopurine and thioguanine, the first being the most used one [13,143]. The largest studies are two open-label trials: one in 18 patients with RCD (10 with RCDI and eight with RCDII) treated with azathioprine at a dose of 2 mg/kg/day after induction with prednisolone [144] and the other in 10 patients with RCDI treated with thioguanine 0.3 mg/kg/day [145]. A clinical and histologic response has been described in 80% of patients, with a 50% decrease in corticosteroids dependency [143,144,145]. If the patient does not respond to immunomodulators, an open-label therapeutic trial on 10 RCDI patients treated with mesalamine alone or in association with budesonide [146], as well as case reports of patients treated with biologics such anti-tumor necrosis factor-α infliximab [147,148,149], have suggested benefit. An open-label trial in 10 patients with RCD (two with RCDII) treated with recombinant human IL-10 failed to show significant benefit, with only three patients presenting a clinical response and two patients a histologic response, who relapsed after IL-10 withdrawal. Furthermore, two patients interrupted the treatment due to side effects (thrombocytopenia and headache) [150].

The current recommendation for RCDI therapy is to start with open-capsule budesonide for at least 3 months, since it is safe and with a high rate of clinical response. After a response, start on azathioprine (at the dose of 2–2.5 mg/kg/day) and evaluate the histologic response at 3 months. After 2–3 years of remission, withdrawal of azathioprine could be considered [14].

For patients with RCDII, immunomodulators should be avoided, due to the lack of efficacy and a potential concern of accelerated lymphoma development [144]. Chemotherapy regimens used to treat EATL are not effective in RCDII because the neoplastic aberrant LIEs are not proliferative [53]. In RCDII patients, the most accepted regimen is purine analogues (cladribine, pentostatin or fludarabine) monotherapy or with sequential autologous stem cell transplantation (ASCT). 

Cladribine (2-chlorodeoxyadenosine, 2-CDA) is a synthetic purine nucleoside analogue, which accumulates in a triphosphate metabolite in cells that highly express deoxycytidine kinase, such as lymphocytes and monocytes [151], promoting cytotoxicity by inducing apoptosis, necrosis and inhibition of DNA/RNA synthesis [152]. Importantly, cladribine is toxic for both proliferating and non-dividing cells [152], it being an advantage to target aberrant IELs that show a low proliferative index. Cladribine has a good safety profile, the most significant side effects being persistent and profound lymphopenia (particularly CD4+ T cells), transient monocytopenia and a modest decrease in platelets, granulocytes and red cell count [153]. A small pilot study evaluated 17 RCDII patients treated with cladribine 0.1 mg/kg intravenously for 2 h daily for 5 consecutive days, with 1 to 3 courses every 6 months, depending on the response [154]. Concomitant prophylaxis of *Pneumocystis jirovecii* pneumonia was performed with cotrimoxazole. The results were clinical improvement in 36% of patients, histologic improvement in 58% and a decrease in aberrant IEL in 35%. Unfortunately, 40% of the patients still developed EATL and died after 14 months on average but as soon as 6 months. Not achieving a decrease in aberrant IEL seemed to associate with EATL development, since only one in seven patients that developed EATL responded with a decrease in aberrant IELs, and that patient had a particularly high baseline aberrant IEL proportion of 73% [154]. Afterwards, the same group presented an extended cohort of 32 RCDII patients treated with cladribine, corroborating the previous results [155]. Cladribine treatment resulted in clinical response in 80% of patients, histologic response in 50% and immunologic response (that is, a decrease higher than 20% in aberrant IELs) at 2 years. Survival at 5 years was higher in responders: 83% versus 22%. Only 16% of patients developed EATL, which is lower than the 30–50% described in natural history studies [28,30,31]. Another research group with 23 RCDII patients achieved similar responses [29].

An option for patients younger than 65 years old is ASCT. The rationale for ASCT is immunoablation by high-dose chemotherapy, with subsequent regeneration of naïve T lymphocytes derived from reinfused hematopoietic progenitor cells [156]. One pilot study on seven RCDII patients achieved engraftment in all patients, resulting in endoscopic resolution and downstaging of Marsh in a histology in six of seven patients, and a 50% decrease in aberrant T cells, 3 to 4 months after transplantation [157]. Subsequently, the same group published a cohort of 18 RCDII patients, of whom, in 13, ASCT was possible, showing a 4-year survival of 100% compared to 23% in the non-transplanted patients. One patient developed EATL after 4 years, suggesting that this approach might delay EATL development as compared to monotherapy with cladribine [158,159].

A staging system has been proposed to help guide the management of RCDII [28]. It consists of the evaluation of the presence of five factors: albumin ≤ 3.2 g/dL, hemoglobin ≤ 11 g/dL, age ≥ 65 years-old, T-cell clone and villous atrophy Marsh 3c. Stages are defined according to the number of factors: stage I, 0–1 point; stage II, 2–3 points; and stage III, ≥4 points. Patients who do not respond to cladribine, or, even if they respond but are classified as stage II or III, should be considered for ASCT [28].

Two drugs, AMG714, an IL-15 inhibitor, and tofacitinib, an oral JAK1/JAK3 inhibitor, were promising, but preliminary results were disappointing since they failed to induce a decrease in aberrant IELs [160,161].

IL-15, a cytokine that specifically promotes the expansion and survival of aberrant IELs, is massively expressed in the mucosa of RCDII patients [48]. As such, the results of a phase 2a 10-week, placebo-controlled randomized trial of AMG-714, a humanized anti-IL-15 monoclonal antibody, on 28 RCDII patients was highly awaited [160]. AMG714 was administered in seven doses of 8 mg/kg each, on day 0, day 7, day 14 and, thereafter, every 2 weeks. AMG714 was safe, with a similar rate of adverse effects compared with placebo. Clinical improvement was unimpressive, though, with a mild improvement in diarrhea, and there were no differences compared with placebo regarding histologic or aberrant IELs improvement. There were, however, benefits regarding IEL TCR clonality, with 100% stable or decreased TCR clonality versus 50% in the placebo group [160].

Tofacitinib, an oral JAK1/JAK3 inhibitor, is under evaluation in a phase 2 open-label trial (NTR 7529, Eudra-CT 2018-001678-10), and aims to target the recurrent JAK1 or STAT3 gain-of-function mutations. The preliminary results on six RCDII patients treated with 10 mg twice daily for 12 weeks were recently published [161]. Clinical improvement (diarrhea, abdominal pain and weight gain) was observed early on, after just 2 days to 2 weeks. However, the symptomatic recurrence was also observed early on and, although four out of six patients improved their histology, it did not result in an immunologic response (absolute decrease of more than 20% of aberrant IELs). This suggests a functional effect in aberrant IELs rather than the induction of apoptosis [161]. Regarding adverse effects, the most common was lymphopenia, but, of concern, one patient developed a pulmonary embolism in the context of a catheter sepsis. Tofacitinib is, therefore, a potential treatment for patients who fail to respond clinically to open-capsule budesonide.

## 5. Conclusions

RCD is a rare condition and occurs in only one out of 10 patients with NRCD. It occurs as two very different entities, RCDI and RCDII. 

Regarding the diagnosis, the main pillars are to exclude gluten contamination of the diet as well as other causes of villous atrophy. Afterwards it is crucial to distinguish between RCDI and RCDII, through the quantification of aberrant IELs. Lastly, particularly in cases of RCDII, complications such as jejunoileitis and overt lymphoma must be thoroughly investigated.

RCDI is a mild condition that probably reflects the progression to an autoimmune intestinal mucosal inflammation that has become independent of gluten exposure. RCDI responds well to immunosuppression and the prognosis is good.

RCDII is a dismal condition, an epithelial low-grade lymphoma. Neoplastic cells are aberrant IELs, whose differentiation in T cells was interrupted by outstanding IL-15 stimulation, acquiring an intermediate phenotype with NK cells. Those neoplastic cells are highly cytotoxic to the mucosal, inducing severe injury and severe clinical malabsorption. Also, aberrant IELs acquire mutations in the JAK1-STAT3 pathway that give them a survival advantage and promote progression towards EATL.

Currently, no therapy can cure RCDII or prevent EATL development, even though a sequential treatment with cladribine and ASCT can induce clinical and histologic responses and possibly delay EATL development. Future therapies that target and eliminate aberrant T cells are needed, as the persistence of those non-dividing cells might function as a reservoir for clinical relapse and fuel EATL development.

## Figures and Tables

**Table 1:** Differences from type 1 and type 2 refractory celiac disease (RCD).

	Type 1 RCD	Type 2 RCD
Female/Male	3:1	1:3
Manifestations	Milder phenotype	Worse abdominal pain Worse malabsorption Worse hypoalbuminemia
Endoscopy	Distal involvement < 20% Ulcerative jejunitis < 30%	Distal involvement 50% Ulcerative jejunitis 67%
Histology	Indistinguishable from CD May present collagenous sprue	Marsh 3c, extensive IEL Aberrant IEL > 50%
Flow cytometry	Aberrant IEL < 20%	Aberrant IEL ≥ 20%
TCR rearrangements	Polyclonal	Monoclonal
Treatment	Glucocorticoids (budesonide) Immunosuppressors (azathioprine) Biologics (anti-TNF-α)	Glucocorticoids (budesonide) Purine analogues (cladribine) ASCT
EATL development	0–14%	30–50%
5-year survival	80–90%	40–50%

ASCT, autologous stem cell transplantation; CD, celiac disease; EATL, enteropathy-associated T-cell lymphoma; IEL, intraepithelial lymphocytes; RCD, refractory celiac disease; TCR, T-cell receptor.

## Abbreviations

ASCT, autologous stem cell transplantation (ASCT); CD, celiac disease; CVID, common variable immunodeficiency; EATL, enteropathy-associated T-cell lymphoma; EMA; anti-endomysium antibody; GFD, gluten free diet; IL, interleukin; JAK, Janus kinase; NRCD, non-responsive celiac disease; pVA, persistent villous atrophy; RCD, refractory celiac disease; RCDI, type I refractory celiac disease; RCDII, type II refractory celiac disease; SOCS-1, suppressor of cytokine signaling-1; STAT, signal transducer and activator of transcription; TCR, T-cell receptor; TLR, toll-like receptor; TTG, anti-transglutaminase-2 antibody.
